# Identification of Metabolic Pathways and Hub Genes Associated with Ultrasound Subcutaneous Fat and Muscle Depth of the Longissimus Muscle in Cull Beef Cows Using Gene Co-Expression Analysis

**DOI:** 10.3390/ani15172636

**Published:** 2025-09-08

**Authors:** Harshraj Shinde, Kyle R. McLeod, Jeffrey W. Lehmkuhler

**Affiliations:** 1 Department of Animal and Food Sciences, University of Kentucky, Lexington, KY 40546, USA; kyle.mcleod@uky.edu (K.R.M.); jeff.lehmkuhler@uky.edu (J.W.L.); 2 Department of Microbiology, Molecular Genetics and Immunology, University of Kansas Medical Center, Kansas City, KS 66103, USA

**Keywords:** cow, beef production, body weight, transcriptome, WGCNA

## Abstract

Beef production is an important component of the world’s food supply, with production being near 59 million tons in 2023 (USDA, 2023). Enhancing our understanding of the factors influencing metabolism will lead to improvements in production efficiency. Using RNA-seq and WGCNA of longissimus muscle samples, gene expression and metabolic pathway analyses were performed to examine relationships with ultrasound and body mass variables. In this study, body weight (BW), ultrasound back fat (BF), ultrasound muscle depth (MD), and body condition score (BCS) were traits recorded for 18 cull beef cows. As expected, all production-related traits monitored (WT, BF, MD, and BCS) in this study exhibited a positive correlation with each other. Large-scale transcriptome analyses were performed using RNA extracted from longissimus dorsi muscles. Weighted correlation network analysis (WGCNA) was employed to associate changes in traits with gene expression. In WGCNA, the dark-green module demonstrated a positive correlation (cor) with all traits, with the highest observed for BF (cor = 0.45, *p* = 0.07) and MD (cor = 0.45, *p* = 0.07). Functional analysis of the dark-green module highlighted olfactory transduction (*p* = 0.03) and RNA processing as significantly correlated (*p* = 0.08) with production traits. Additionally, the hematopoietic cell lineage pathway was reported as the most significant negative correlation with muscle depth (cor = −0.71, *p* = 0.001). We identified four hub genes (i.e., SEPTIN9, NONO, CCDC88C, and CACNA2D3) showing relationships with the traits measured. These findings provide further understanding of the molecular mechanisms influencing muscle and fat accretion in cull beef cows.

## Simple Summary

Our current transcriptomics analyses aimed to improve understanding of how genes influence muscle and fat growth in beef cows. As the demand for good quality beef continues to increase, finding ways to improve and understand mechanism influencing beef quality is important. By employing transcriptomics analysis and WGCNA based gene co-expression analyses, we examined the relationship between candidate genes and various beef quality traits like body weight (BW), ultrasound back fat (BF), ultrasound muscle depth (MD) and body condition score (BCS) in beef cows. Our results highlighted interesting candidate genes and metabolic pathways associated with these traits, providing valuable insights into the molecular mechanisms that control muscle and fat growth. By understanding these mechanisms, farmers and producers can develop more effective strategies to improve beef production, ultimately benefiting both the industry and consumers. This research contributes to the ongoing efforts to enhance the sustainability and efficiency of beef production. Our study reveals novel insights into the biological processes that influence muscle and fat growth, paving the way for future research and efficient beef production.

## 1. Introduction

Beef is an excellent source of protein, and its demand is increasing globally [1]. Beef production efficiency is directly linked to profitability and sustainability of the beef cattle industry [2]. Beef production is a complex system and influenced by different factors such as age, breed, diet, genetic factors (genes), weather, genotype-by-environment interaction and other factors [3,4]. Most of the genetic or molecular factors involved in beef production are not completely understood. Few genes related to beef production in cows have been reported. For example, ACACA (Acetyl-CoA carboxylase alpha) gene is reported as a major player in intramuscular deposition in ruminants. In beef cows, a positive correlation between *ACACA* expression level and total lipids and trans-fatty acids were reported [5]. Pleomorphic adenoma gene 1 (PLAG1) and Pleomorphic adenoma gene 2 (PLAG2) were reported to be associated with growth, stature, skeletal muscle growth, fat thickness, and fatty acid composition in cattle [6]. A haplotype analysis found evidence that the mutation in PLAG1 mutation affects body size and weight in modern cattle [7].

Next-generation sequencing allows detailed analysis of complex traits at the level of their genomes (genomics), transcriptomes (transcriptomics), or epigenome (epigenomics) [8]. Among these, transcriptomics is widely utilized to identify regulators of complex traits at gene/transcript level. Ribonucleic acid (RNA) sequencing (RNA-Seq) is a popular approach for transcriptome profiling that uses next-generation sequencing analysis. RNA-Seq gives detailed quantification of transcripts, and their isoforms compared to other techniques [9]. RNA-Seq has been utilized for studying gene expression profiles of different animals, e.g., cow, horse, sheep, buffalo and others [10,11,12,13]. Most of these studies have identified differentially expressed genes and their associated pathways. However, additional identification of genes and their relationship to important traits can shed more light on how genes regulate complex traits. Weighted Gene Co-Expression Network Analysis (WGCNA) is a widely used technique for co-expression network analysis to screen gene expression data and relationships with phenotypic traits [14]. WGCNA has been widely used to study complex traits and their correlation with gene expression data [15]. In cows, WGCNA has been used to investigate feed efficiency. One study identified *ATP7B* (ATPase Copper Transporting Beta) as a hub gene (central regulator) significantly associated with residual feed intake [15]. In Nellore cattle, relationships of between gene expression and carcass traits were revealed using WGCNA [14]. Authors reported that energy and lipid metabolism pathways, highlighting fatty acid metabolism, were the central pathways associated with rib-eye area.

In an effort to further understand the complex relationships of production traits and gene expression in beef cows, we employed RNA-Seq based transcriptome data and WGCNA. In our study, body weight, body condition score, back fat and muscle depth were traits (variables) associated with beef production which were monitored. Total RNA was extracted from the *longissimus dorsi* with RNA-Seq analyses conducted. Weighted gene co-expression network analysis was applied to associate changes in beef production related traits with gene expression. Relationships between measured traits, metabolic pathways, and hub genes were then identified.

Despite growing use of RNA sequencing and co-expression analyses in animal research, the gene networks and key regulators associated with muscle development and fat deposition in beef cows remain poorly understood. Especially, the integration of transcriptome profiling with phenotypic traits using WGCNA. To address this gap, we analyzed gene co-expression modules and hub genes associated with beef production traits in cows using RNA sequencing and WGCNA. Our hypothesis is that identified gene modules are significantly correlated with economically relevant traits and that hub genes within these modules may serve as key regulators of muscle growth and fat accumulation. Our research approach helps to fill a critical gap in the current understanding of the molecular mechanisms underlying beef production efficiency.

## 2. Materials and Methods

### 2.1. Experimental Site

All the procedures were conducted according to a protocol approved by the Institutional Animal Care and Use Committee at the University of Kentucky (protocol #2022-4039). The facility was inspected and approved by the Institutional Animal Care and Use Committee. Animals were individually penned (2.4 m × 14.6 m pens) in a partially covered concrete floored pen. The covered area was bedded with sawdust. Each pen contained an automatic waterer shared between two pens. Feed was delivered in concrete bunks each morning.

### 2.2. Beef Production Related Trait Analysis

Twenty-two mature cows of unknown age and genetic background were purchased from local markets between 23 May and 6 June 2022. Most had black haircoats, suggesting Angus influence, while others showed Charolais or Hereford traits. Body condition scores and weights were recorded on 10 June. Three cows were later removed due to health issues, and one Angus cow from the university herd was added, resulting in 20 cows for the study. After arrival, cows received hay ad libitum, then were transitioned to a limit-fed diet of corn silage and protein supplement at 1.5× maintenance energy requirements.

### 2.3. Longissimus Dorsi Muscle Biopsy

Muscle biopsies of the *Longissimus dorsi* were performed between the 12th and 13th ribs on 29 July 2022. Ultrasonography guided sampling by targeting the mid-point of the muscle to ensure consistency across animals. Biopsies were taken from the right side after clipping and disinfecting the area. A local injection of 5 mL lidocaine (2%) was administered before using a 14-gauge, 2.54 cm needle for incision. Approximately 200 mg of muscle tissue was collected using a semi-automatic biopsy needle (Argon based in Frisco, TX, USA), and visible fat was removed. Samples for RNA extraction were preserved in RNAlater™, (Thermo Fisher Scientific, Waltham, MA, USA, RNAlater™ Stabilization Solution; Catalog No. AM7020) flash frozen in liquid nitrogen, and stored at −80 °C.

### 2.4. RNA Extraction, Library Preparation and Sequencing

Total RNA was extracted from muscle samples using the RNA MicroPrep Kit (Zymo Research, Irvine, CA, USA). RNA quality was examined by Bioanalyzer (Agilent 2100, Santa Clara, CA, USA), samples showing RIN values > 8.0. were further processed. The cDNA libraries were prepared using the KAPA mRNA-Seq Kit (Roche, Basel, Switzerland, KR0960). Libraries were size-selected, PCR-amplified, quality-checked and sequenced using Illumina NovaSeq S4 platform (2 × 150 bp, paired-end). All procedures were conducted by Quick Biology Inc. (Pasadena, CA, USA)

### 2.5. RNA Sequencing Data Analysis

**Figure 1:**
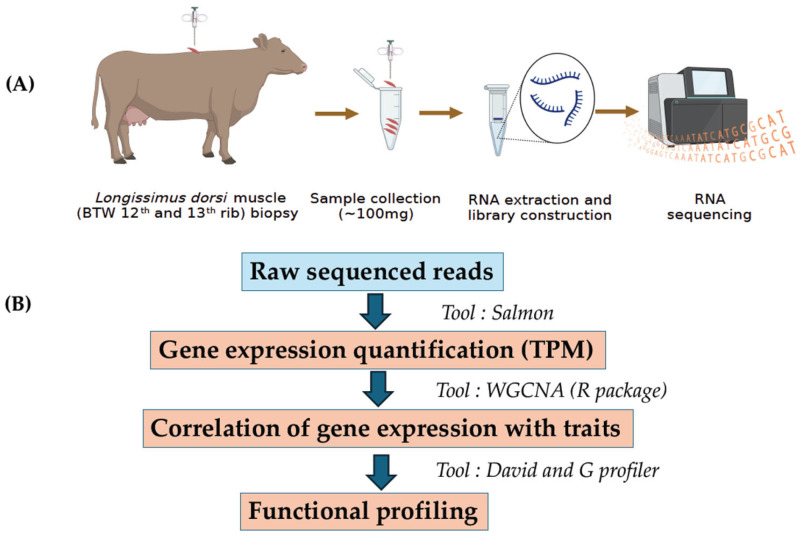
The workflow of RNA sequencing and co-expression analysis includes two parts: (**A**) Sample collection, RNA extraction, library preparation, and sequencing. (**B**) Processing the raw sequencing reads, quantification of gene expression profile, correlation of gene expression with traits, and functional profiling.

Raw sequencing reads (FASTQ files) were subjected to quality control by FastQC https://www.bioinformatics.babraham.ac.uk/projects/fastqc/ (accessed on 1 August 2022). In brief, FastQC detected contaminants including overrepresented sequences, sequencing adapters, poor quality reads and PCR duplicates. All these contaminants were filtered using Cutadapt 1.4 (Command line: cutadapt -a ADAPTER_FWD -A ADAPTER_REV -o out.1.fastq -p out.2.fastq reads.1.fastq reads.2.fastq) [16,17]. The cow reference transcriptome files (Bos taurus ARS_UCD 1.2) for mapping and quantification were obtained from Ensembl genome browser https://ftp.ensembl.org/pub/release-108/fasta/bos_taurus/cdna/ (accessed on 10 August 2022). The initial step involved indexing the cow reference transcript file using the Salmon index option. (command line: salmon_index -t cow_transcripts.fasta -d decoys.txt -p 12 -i cow_salmon_index). The transcriptome-wide quantification in the form of TPM (transcript per million) on clean reads were performed using salmon v 1.6.0 (command line: salmon quant -i salmon_index -l A -1 Clean_Read_1.fq -2 Clean_Read_2.fq --validateMappings -o salmon_quant -p 12 --seqBias) [17]. The workflow of RNA sequencing and data analyses are shown in Figure 1.

### 2.6. Weighted Gene Co-Expression Network Analysis (WGCNA)

The R package, Weighted Gene Co-expression Network Analysis (WGCNA) was used to construct co-expression networks and find co-expressed genes [14]. Gene expression data (TPM) of 18 cows with four different traits (WT, BF, MD and BCS) were selected for WGCNA. Two cows were removed due to being outliers. At first, the low count genes and outliers were filtered. To choose modules associated with traits (WT, BF, MD and BCS) of our interest, Pearson correlation analysis between each module’s “eigengene” and the traits were determined. Modules are groups of genes with similar expression profiles and tend to be functionally related and co-regulated. Modules with a module-trait correlation >0.4 or <−0.4 for at least one trait (*p* ≤ 0.10) were considered significant. Gene significance (GS) was calculated for each gene as the correlation between gene expression counts and traits (WT, BF, MD and BCS). Hub genes were identified by choosing genes with high gene significance and module membership in the modules of interest.

### 2.7. Functional Enrichment Analysis

Associated modules were selected (R > 0.4 or <−0.4, *p* ≤ 0.10) for functional enrichment analysis. Here “R” refers to the Pearson correlation coefficient, which quantifies the linear relationship between module eigengenes and external traits. KEGG pathways and gene ontology terms enriched in these modules were identified using gprofiler interface https://biit.cs.ut.ee/gprofiler/ (accessed on 10 September 2022) with threshold of a Benjamini–Hochberg FDR (false discovery rate) of 0.05 [18]. We chose the “g:GOSt—Functional profiling of gene lists” option for conducting the metabolic pathway and gene ontology analysis within the selected *Bos taurus* reference database. The graphical representation of most significant pathways was achieved using R package (version 4.2.1) ggplot2 https://ggplot2.tidyverse.org/index.html (accessed on 15 September 2022) [17].

## 3. Results

### 3.1. Trait Relationship Analysis

**Figure 2:**
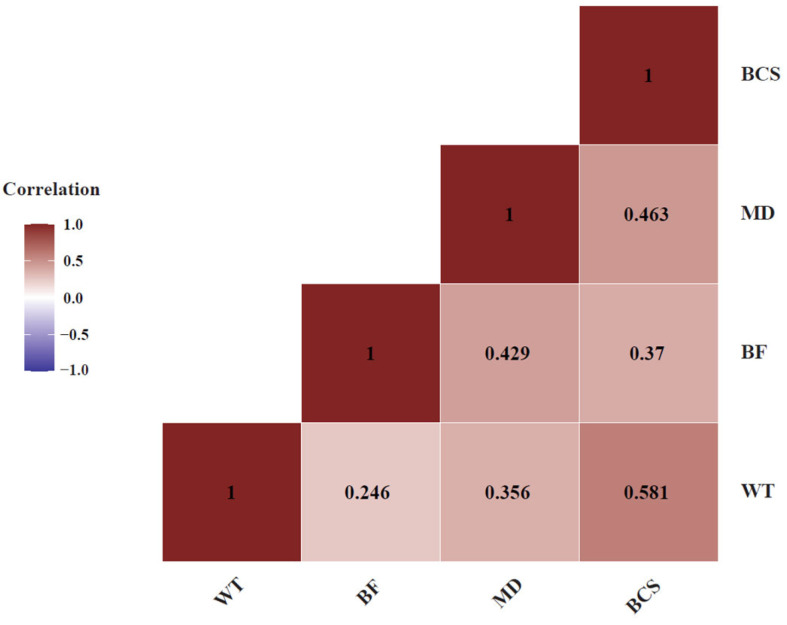
Pearson correlation analysis between four beef production related traits: body weight (BW), back fat (BF), muscle depth (MD) and body condition score (BCS). The traits mostly exhibited positive correlations with each other.

We recorded four traits in these cows (Table 1). Covariation between these four traits showed that all four traits are positively correlated with each other as expected. Among these traits, BCS and WT had a correlation of 0.581, whereas MD and BCS were also positively correlated, at 0.463 (Figure 2).

### 3.2. RNA Sequencing

RNA sequencing yielded an average of 33 million raw reads per sample. On average, 98.80% of the raw reads successfully passed the quality check. The average percentage of mappable clean reads per sample were 70.13%, ranging from 64.3 to 74.7% (Supplementary File S1). The transcript per million (TPM) for all 37,926 cow genes were estimated, the average TPM per sample was 26 (Supplementary File S2). Out of 37,926 genes, 7655 genes were removed from further analysis due to excessive missing samples or zero variance.

### 3.3. Weighted Gene Co-Expression Network Analysis (WGCNA)

**Figure 3:**
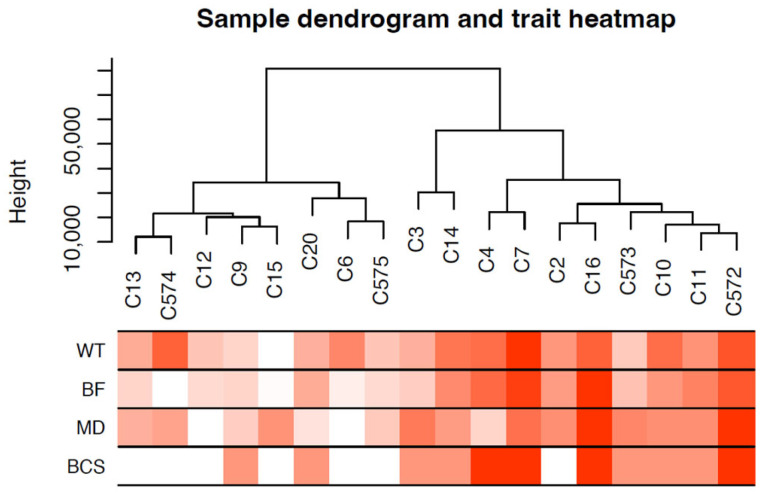
Sample dendrogram and trait heatmap. The dendrogram plotted by hierarchical clustering using gene expression of 18 cow samples. The heatmap below the dendrogram indicates trait data. The analysis clearly divided the 18 cows into two groups: light-weight cows (8 cows) and heavyweight cows (10 cows).

**Figure 4:**
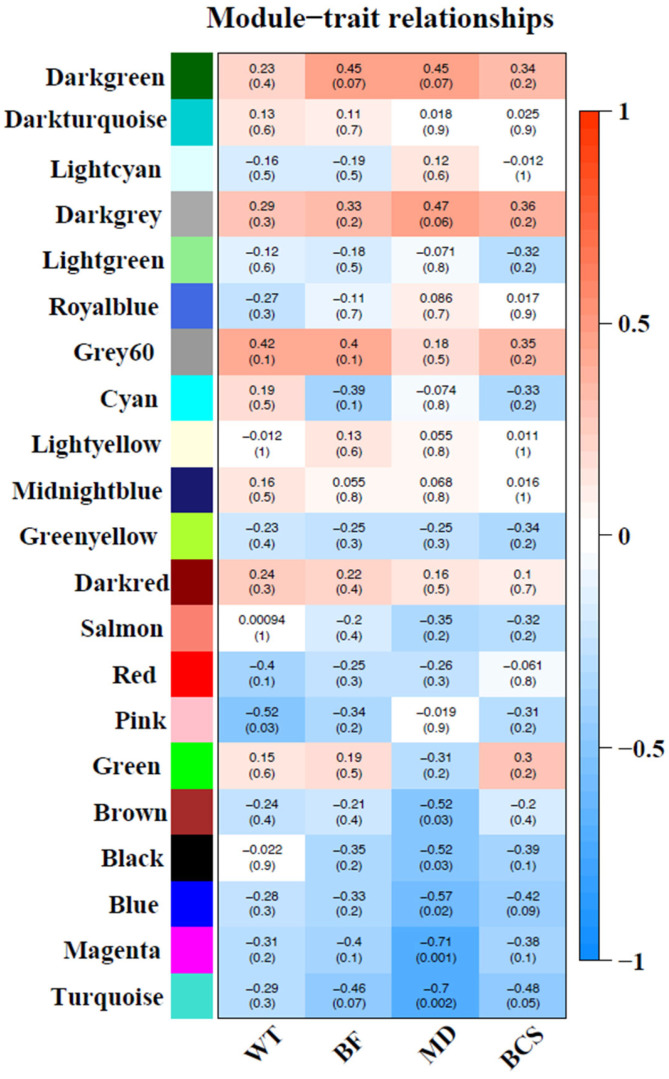
Correlation between modules (group of genes) and traits [body weight (BW), back fat (BF), muscle depth (MD) and body condition score (BCS)]. Module names are displayed on the left, and the correlation coefficients are shown at the top of each row. The *p*-values for each module are displayed at the bottom of each row within parentheses. The rows are colored based on the correlation of the module with the trait: Red for positive and blue for negative correlation.

Sample dendrogram produced by hierarchical clustering is shown in Figure 3. Sample dendrogram based on gene expression and heatmap using trait data clearly divided the 18 cows into two groups: group 1—with mostly light weight cows (8 cows) and group 2—mostly cows having heavier live weights (10 cows). The WGCNA constructed 21 modules (i.e., cluster of co-expressed genes) identified by a different color. The WGCNA also yielded correlations between the genes of each module and measured traits (WT, BF, MD and BCS). Figure 4 illustrates the association between modules and traits, which represents Pearson’s correlation coefficients measured between each single module and trait. Given the vast amount of data, we focused on two modules, i.e., dark-green and magenta, which showed significant positive and negative correlation with all four traits. Data for all genes, their respective modules (21 eigengene modules), and correlation values are given in Supplemental File S3.

### 3.4. Functional Enrichment Analysis

**Figure 5:**
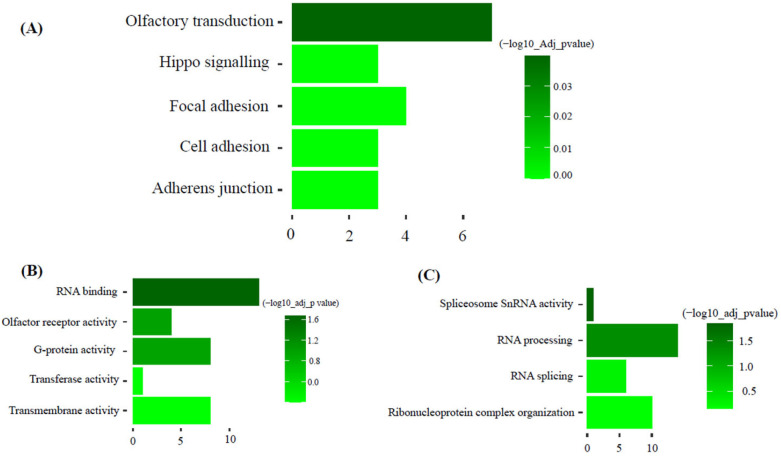
Functional enrichment analysis of dark-green module. (Top five pathways or terms shown). (**A**): KEGG: Metabolic Pathways. (**B**): GO: Molecular function. (**C**): GO: Biological process.

**Figure 6:**
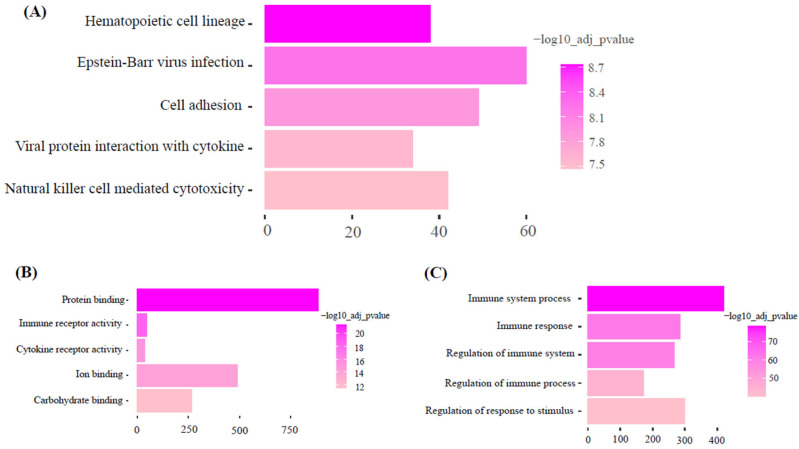
Functional enrichment analysis of Magenta module. (Top five pathways or terms shown). (**A**): KEGG: Metabolic Pathways. (**B**): GO: Molecular function. (**C**): GO: Biological process.

KEGG pathway analysis of the dark-green module showed ‘olfactory transduction’ as the most significant pathway. Gene ontology molecular function and biology process analysis of dark-green showed RNA binding and spliceosome snRNA activity as significantly enriched GO terms (Figure 5). Additionally, KEGG pathway analysis of the magenta module showed hematopoietic cell lineage as the most significant pathway. Gene ontology molecular function and biology process analysis of magenta showed protein binding and immune system process as significantly enriched GO terms (Figure 6).

### 3.5. Hub Gene Identification

**Figure 7:**
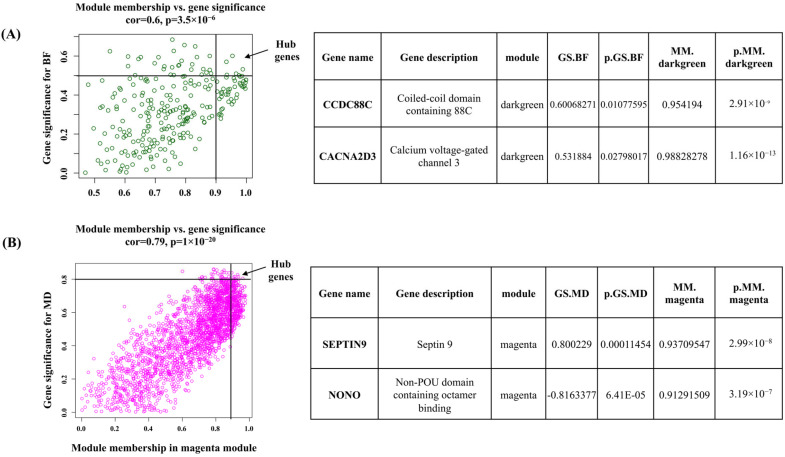
Hub genes determined by module membership (MM) and gene significance (GS). GS represents the correlation between a gene and a trait. The MM represents the correlation between an individual gene and the module eigengene. MM and GS values were plotted on the x and *y*-axis. (**A**) In dark green module hub genes were identified according to a GS > 0.5 and an MM > 0.9. (**B**) In Magenta hub genes were identified according to a GS > 0.8 and an MM > 0.9. Table represent the top two hub genes of individual module.

For the dark-green module, *CCDC88C* (Coiled-coil domain containing 88C) and *CACNA2D3* (Calcium voltage-gated channel 3) were identified as hub genes. These genes have gene significance > 0.5 with back fat and module membership >0.9 with dark-green module eigengene. In the magenta module, *SEPTIN9* and *NONO* (Non-POU domain containing octamer binding) genes were defined as hub genes based on their gene significance >0.8 with muscle depth and module membership >0.9 with magenta module eigengene (Figure 7).

## 4. Discussion

### 4.1. Overview of Transcriptomics and Gene Co-Expression Analysis

The objective of the study was to identify candidate genes and metabolic pathways that could provide a genetic insight into tissue accretion of cull beef cows. We performed transcriptomic analyses of *longissimus dorsi* muscle. Using gene expression data from the *longissimus dorsi* muscle tissue, gene co-expression modules were built and correlated to traits associated with beef production. Our study stands apart and contributes novel insights compared to previous research in related livestock species as, most of the previous transcriptomic studies in cattle focus on growing steers, heifers, or dairy breeds. Our study uniquely targets cull beef cows, a population often overlooked despite its relevance to carcass quality and economic value in the beef industry. As differential expression analysis is common approach, our study applies WGCNA to identify gene modules, hub genes and metabolic pathways associated with muscle and fat traits. WGCNA identified gene modules, metabolic pathways, and candidate genes that are expected to have a biological role in beef production. Out of 21 gene modules, the modules having the greatest significant correlations to the traits measured were further analyzed and discussed.

### 4.2. Functional Insights from the Dark-Green Module

Among these modules, the Dark-green module (356 genes) had a significant positive correlation with back fat and muscle depth. The functional enrichment analysis highlighted olfactory transduction as a significant pathway and RNA activity (processing, splicing, and binding) as a significant GO term. In the olfactory transduction pathway, odorant or food is detected with signals sent to the brain or other body parts [19]. These signals are, in general, influenced by calcium, cyclic AMP and G protein molecules. In animals, olfactory transduction is directed linked with appetite, feed preference and weight gain [20,21]. In the pig, olfactory transduction pathway exhibited direct association with residual feed intake [22]. In beef cattle (SimAngus), quantitative trait loci detected for residual feed intake was flanked by eight olfactory receptor genes [23]. Thus, biologically animals with an upregulation of feed intake would be expected to have increased tissue accretion or at least for the traits measured in this study.

Another noteworthy pathway found in the dark green module is the hippo signaling pathway. Previous reports have shown that the hippo signaling pathway regulates the expression of Yes-associated protein (Yap) and transcriptional co-activator with PDZ-binding motif (Taz). Both Yap and Taz genes are critical regulators of skeletal muscle mass. Additionally, these genes are involved in controlling tissue growth in various cell types [24].

### 4.3. Role of RNA Processing in Muscle Development

The significant gene ontology terms of dark-green modules were RNA binding, snRNA activity, RNA processing and RNA splicing. All these terms come under RNA processing and modifications. According to previous literature, muscle development relies on RNA processing. It is mainly driven by coordination among RNA binding proteins. Additionally, misregulation of RNA processing causes muscle diseases [25,26]. Alternative splicing is a mechanism that enables single genes to produce multiple mature mRNAs. Among various tissues, skeletal muscle cells, along with the brain and heart, display the most prominent levels of tissue-specific and evolutionarily conserved alternative splicing events. These molecular alterations are accompanied by a multitude of transcriptional and posttranscriptional changes, many of which are governed by alternative splicing mechanisms [25,27,28,29]. In mice, RNA splicing of the Fragile-X mental retardation autosomal homolog-1 (FXR1) gene directly contributes to muscle development. The FXR1 gene is hypothesized to play a regulatory role in mRNA translation, localization, and stability within muscle cells [30]. Thus, cull beef cows which are emaciated due to restricted nutrient intakes and then provided an increased plane of nutrition may elicit responses for protein accretion.

### 4.4. Functional Roles of Magenta and Turquoise Modules

The magenta and turquoise modules had negative correlation with back fat, muscle depth and body condition score. The functional analysis of magenta highlighted hematopoietic cell lineage pathway as the most significant pathway. In animals, tissue self-renew, tissue growth and muscle tissue regeneration are mainly controlled by Hematopoietic cell lineage [31]. During muscle growth, Hematopoietic cell lineage pathway is activated. However, in the current study the opposite was observed showing less expression for increasing weight, BCS, MD and BF. Detailed characterization of this pathway is necessary to study the exact role of hematopoietic cell lineage in beef production. It is possible this response observed in this group of cull beef cows is an artifact of previous management in which nutrient intake was less than the animal’s requirements. In regard to the turquoise module, it was noted that the oxidative phosphorylation pathway was the most highly significant pathway. Oxidative phosphorylation provides energy in the form of ATP to support muscle growth and maintenance [32]. In buffalo, the WGCNA identified pathways related to energy metabolism, such as oxidative phosphorylation, that play a role in fat deposition [33]. Thus, if animals were in a negative energy state previously, it would be plausible the expression of genes in this pathway may suppressed. Supporting this, multi-omics studies in Nellore cattle have demonstrated that genes related to muscle growth, energy metabolism, and fat deposition like FRZB, IGFBP5, CAND1, and NCOR2 are expressed in animals with divergent carcass and meat quality traits. Particularly, oxidative phosphorylation-related genes were implicated in variations in meat tenderness and fat-related traits. These findings align with our current results and highlight the importance of mitochondrial and structural pathways in determining carcass composition [34].

### 4.5. Hub Genes in the Dark-Green Module

Hub genes are candidate genes which are important in the regulation of the expression of several other genes in a module and are potential biomarkers for the selection of a trait [35]. Such an approach has already been employed in ruminants to identify genes governing traits [36,37,38,39,40]. The WGCNA in buffalo identified six hub genes [FH (Fumarate Hydratase), MECR (Mitochondrial Trans-2-Enoyl-CoA Reductase), GPI (Glucose-6-Phosphate Isomerase), PANK3 (Pantothenate Kinase 3), ATP6V1A (ATPase H+ Transporting V1 Subunit A), PHYH (phytanoyl-CoA Hydroxylase)]. These hub genes were postulated to have associations with a range of aspects, encompassing growth and development, fat deposition, and the levels of amino acids within muscle tissue in buffalo [33]. In the dark-green module, CCDC (Coiled-coil domain containing 88C) and CACNA2D3 (Calcium voltage-gated channel 3) were reported as central hub genes. Calcium voltage-gated channel is regulated by calcium and responsible for muscle contraction [41]. The calcium channels also supply calcium ions to muscles, and these ions serve as secondary messengers, governing cellular functions within muscle tissue [42].

### 4.6. Hub Genes in Magenta and Turquoise Modules

In magenta module, SEPTIN9 and NONO (Non-POU domain containing octamer binding) were identified as the most significant hub genes. Septin 9 is a cell cycle-related gene and indispensable for coordinating myosin motor proteins during cytokinesis [43,44]. In animals, the NONO gene is a multifunctional gene involved in transcription regulation and alternative splicing. Notably, in mice, the NONO gene plays a direct and critical role in B cell development through the ERK (Extracellular Signal-Regulated Kinase), AKT (PI3K/AKT/mTOR), and NF-κB (Nuclear Factor-κB) pathways [45]. This underscores the importance of NONO in the intricate process of cellular and muscle development in animals [45]. Further experiments are necessary to validate the modules, metabolic pathways, and hub genes uncovered in the present study.

### 4.7. Study Limitations

Our study provides valuable insights into the genetic mechanisms underlying tissue accretion in cull beef cows being fed to regain body mass prior to harvesting, certain limitations should be acknowledged. First, the cows used in our study were of unknown age and genetic background which may have introduced variability in our gene expression and phenotypic data. The smaller sample size may affect statistical power and the generalizability of the results. Moreover, previous nutritional or management histories of the cows may have introduced confounding variables. Although transcriptomic analyses with WGCNA enabled the identification of gene modules, pathways, and hub genes, the absence of downstream functional validation studies warrants caution on the application of the outcomes in the present study. Future studies incorporating larger sample sizes, genetic characterization, and functional validation of genes will be necessary to substantiate these findings. The present study did not study epigenetic mechanisms such as DNA methylation, histone modifications, or non-coding RNAs. These factors are known to largely influence gene expression, particularly in response to nutrition. Integrating epigenomic data in future studies could provide a more comprehensive understanding of the regulatory networks underlying these traits. The findings presented in this work provide a foundation for future studies in a class of beef animals not widely investigated. Given the United States’ shrinking beef cow herd size, reconditioning cull cows prior to harvest will become more important. Increasing our understanding of the underlying mechanisms that enhance production efficiency of cull cows will allow for increased beef supply from cull cows in the near future.

## 5. Conclusions

Our findings provide novel insights into the transcriptional signature underlying muscle and fat development in beef cattle. The identified modules, hub genes, and key pathways contribute to a deeper understanding of biological responses to fat development in beef cattle and help in the development of genetic markers aimed at improving beef production. Our studies lay a foundation for future studies to improve beef production through genetic and molecular approaches.

## Figures and Tables

**Table 1:** Beef production related traits recorded in this study (18 cows). WT: Body weight, BF: Back fat, MD: Muscle depth and BCS: Body condition score.

Cow_Id	Weight (WT), Kg	Backfat (BF), cm	Muscle Depth (md), cm	Body Condition Score (bcs)
C2	535	0.62	5.02	4
C3	509	0.42	5.32	5
C4	584	0.82	3.96	6
C6	555	0.3	3.29	4
C7	652	0.99	5.45	6
C9	463	0.4	4.08	5
C10	585	0.63	5.05	5
C11	540	0.72	5.05	5
C12	483	0.37	3.34	4
C13	513	0.4	4.53	4
C14	575	0.69	4.83	5
C15	415	0.25	4.93	4
C16	599	1.04	6.41	6
C20	508	0.55	3.74	5
C572	612	0.89	6.44	6
C573	478	0.47	5.12	5
C574	599	0.22	4.68	4
C575	482	0.37	4.14	4
Average	538.16	0.56	4.74	4.8

## Data Availability

Raw sequencing reads generated by RNA sequencing were deposited in the NCBI-SRA database under the bioproject accession number PRJNA933175.

## Supplementary Materials

The following supporting information can be downloaded at: https://www.mdpi.com/article/10.3390/ani15172636/s1, Supplementary File S1: The sequencing statistics of RNA sequencing reads. Supplementary File S2: Transcript per million (TPM) of 18 cow samples. Supplementary File S3: Data of all identified WGCNA modules and their module correlations and membership values. For Muscle depth (MD), back fat (BF), body condition score (BCS) and body weight (WT).

## References

[1] Pighin D., Pazos A., Chamorro V., Paschetta F., Cunzolo S., Godoy F., Messina V., Pordomingo A., Grigioni G. (2016). A Contribution of Beef to Human Health: A Review of the Role of the Animal Production Systems. Sci. World J..

[2] Greenwood P.L. (2021). Review: An overview of beef production from pasture and feedlot globally, as demand for beef and the need for sustainable practices increase. Animal.

[3] Mwangi F.W., Charmley E., Gardiner C.P., Malau-Aduli B.S., Kinobe R.T., Malau-Aduli A.E.O. (2019). Diet and genetics influence beef cattle performance and meat quality characteristics. Foods.

[4] Sakowski T., Grodkowski G., Gołebiewski M., Slósarz J., Kostusiak P., Solarczyk P., Puppel K. (2022). Genetic and Environmental Determinants of Beef Quality—A Review. Front. Vet. Sci..

[5] da Costa A.S.H., Pires V.M.R., Fontes C.M.G.A., Mestre Prates J.A. (2013). Expression of genes controlling fat deposition in two genetically diverse beef cattle breeds fed high or low silage diets. BMC Vet. Res..

[6] Grigoletto L., Ferraz J.B.S., Oliveira H.R., Eler J.P., Bussiman F.O., Abreu Silva B.C., Baldi F., Brito L.F. (2020). Genetic Architecture of Carcass and Meat Quality Traits in Montana Tropical^®^ Composite Beef Cattle. Front. Genet..

[7] Utsunomiya Y.T., Milanesi M., Utsunomiya A.T.H., Torrecilha R.B.P., Kim E.S., Costa M.S., Aguiar T.S., Schroeder S., Do Carmo A.S., Carvalheiro R. (2017). A PLAG1 mutation contributed to stature recovery in modern cattle. Sci. Rep..

[8] Sharma A., Park J.-E., Chai H.-H., Jang G.-W., Lee S.-H., Lim D. (2017). Next generation sequencing in livestock species—A Review. J. Anim. Breed. Genom..

[9] Wang Z., Gerstein M., Snyder M. (2009). RNA-Seq: A revolutionary tool for transcriptomics. Nat. Rev. Genet..

[10] Sood T.J., Lagah S.V., Mukesh M., Singla S.K., Chauhan M.S., Manik R.S., Palta P. (2019). RNA sequencing and transcriptome analysis of buffalo (Bubalus bubalis) blastocysts produced by somatic cell nuclear transfer and in vitro fertilization. Mol. Reprod. Dev..

[11] Chopra-Dewasthaly R., Korb M., Brunthaler R., Ertl R. (2017). Comprehensive RNA-Seq profiling to evaluate the sheep mammary gland transcriptome in response to experimental mycoplasma agalactiae infection. PLoS ONE.

[12] Capomaccio S., Vitulo N., Verini-Supplizi A., Barcaccia G., Albiero A., D’Angelo M., Campagna D., Valle G., Felicetti M., Silvestrelli M. (2013). RNA sequencing of the exercise transcriptome in equine athletes. PLoS ONE.

[13] Yan Z., Huang H., Freebern E., Santos D.J.A., Dai D., Si J., Ma C., Cao J., Guo G., Liu G.E. (2020). Integrating RNA-Seq with GWAS reveals novel insights into the molecular mechanism underpinning ketosis in cattle. BMC Genom..

[14] Langfelder P., Horvath S. (2008). WGCNA: An R package for weighted correlation network analysis. BMC Bioinform..

[15] Salleh S.M., Mazzoni G., Løvendahl P., Kadarmideen H.N. (2018). Gene co-expression networks from RNA sequencing of dairy cattle identifies genes and pathways affecting feed efficiency. BMC Bioinform..

[16] Bolger A.M., Lohse M., Usadel B. (2014). Trimmomatic: A flexible trimmer for Illumina sequence data. Bioinformatics.

[17] Wickham H. (2016). ggplot2 Elegant Graphics for Data Analysis.

[18] Raudvere U., Kolberg L., Kuzmin I., Arak T., Adler P., Peterson H., Vilo J. (2019). G:Profiler: A web server for functional enrichment analysis and conversions of gene lists (2019 update). Nucleic Acids Res..

[19] Su C.Y., Menuz K., Carlson J.R. (2009). Olfactory Perception: Receptors, Cells, and Circuits. Cell.

[20] Ramos-Lopez O., Riezu-Boj J.I., Milagro F.I., Zulet M.A., Santos J.L., Martinez J.A., MENA project (2019). Associations between olfactory pathway gene methylation marks, obesity features and dietary intakes. Genes Nutr..

[21] Riera C.E., Tsaousidou E., Halloran J., Follett P., Hahn O., Pereira M.M.A., Ruud L.E., Alber J., Tharp K., Anderson C.M. (2017). The Sense of Smell Impacts Metabolic Health and Obesity. Cell Metab..

[22] Do D.N., Strathe A.B., Ostersen T., Pant S.D., Kadarmideen H.N. (2014). Genome-wide association and pathway analysis of feed efficiency in pigs reveal candidate genes and pathways for residual feed intake. Front. Genet..

[23] Seabury C.M., Oldeschulte D.L., Saatchi M., Beever J.E., Decker J.E., Halley Y.A., Bhattarai E.K., Molaei M., Freetly H.C., Hansen S.L. (2017). Genome-wide association study for feed efficiency and growth traits in U.S. beef cattle. BMC Genom..

[24] Watt K.I., Goodman C.A., Hornberger T.A., Gregorevic P. (2018). The Hippo Signaling Pathway in the Regulation of Skeletal Muscle Mass and Function. Exerc. Sport Sci. Rev..

[25] Hinkle E.R., Wiedner H.J., Black A.J., Giudice J. (2019). RNA processing in skeletal muscle biology and disease. Transcription.

[26] Shi D.L., Grifone R. (2021). RNA-Binding Proteins in the Post-transcriptional Control of Skeletal Muscle Development, Regeneration and Disease. Front. Cell Dev. Biol..

[27] Bland C.S., Wang E.T., Vu A., David M.P., Castle J.C., Johnson J.M., Burge C.B., Cooper T.A. (2010). Global regulation of alternative splicing during myogenic differentiation. Nucleic Acids Res..

[28] Brinegar A.E., Xia Z., Loehr J.A., Li W., Rodney G.G., Cooper T.A. (2017). Extensive alternative splicing transitions during postnatal skeletal muscle development are required for calcium handling functions. Elife.

[29] Apponi L.H., Corbett A.H., Pavlath G.K. (2011). RNA-binding proteins and gene regulation in myogenesis. Trends Pharmacol. Sci..

[30] Smith J.A., Curry E.G., Eric Blue R., Roden C., Dundon S.E.R., Rodríguez-Vargas A., Jordan D.C., Chen X., Lyons S.M., Crutchley J. (2020). FXR1 splicing is important for muscle development and biomolecular condensates in muscle cells. J. Cell Biol..

[31] Lee J.Y., Hong S.H. (2020). Hematopoietic stem cells and their roles in tissue regeneration. Int. J. Stem. Cells.

[32] Wilson D.F. (2017). Oxidative phosphorylation: Regulation and role in cellular and tissue metabolism. J. Physiol..

[33] Wang S., Yang C., Pan C., Feng X., Lei Z., Huang J., Wei X., Ma Y. (2022). Identification of key genes and functional enrichment pathways involved in fat deposition in Xinyang buffalo by WGCNA. Gene.

[34] Frezarim G.B., Mota L.F.M., Fonseca L.F.S., Salatta B.M., Arikawa L.M., Schmidt P.I., Silva D.B., Albuquerque L.G. (2025). Multi-omics integration identifies molecular markers and biological pathways for carcass and meat quality traits in Nellore cattle. Sci. Rep..

[35] Santri I.N., Irham L.M., Djalilah G.N., Perwitasari D.A., Wardani Y., Phiri Y.V.A., Adikusuma W. (2022). Identification of Hub Genes and Potential Biomarkers for Childhood Asthma by Utilizing an Established Bioinformatic Analysis Approach. Biomedicines.

[36] Keogh K., Kenny D.A., Waters S.M. (2019). Gene co-expression networks contributing to the expression of compensatory growth in metabolically active tissues in cattle. Sci. Rep..

[37] Cánovas A., Reverter A., DeAtley K.L., Ashley R.L., Colgrave M.L., Fortes M.R.S., Islas-Trejo A., Lehnert S., Porto-Neto L., Rincón G. (2014). Multi-tissue omics analyses reveal molecular regulatory networks for puberty in composite beef cattle. PLoS ONE.

[38] Miklos A.C., Li C., Sorrell C.D., Lyon L.A., Pielak G.J. (2011). An upper limit for macromolecular crowding effects. BMC Biophys..

[39] Gu Q., Nagaraj S.H., Hudson N.J., Dalrymple B.P., Reverter A. (2011). Genome-wide patterns of promoter sharing and co-expression in bovine skeletal muscle. BMC Genom..

[40] Hudson N.J., Reverter A., Wang Y.H., Greenwood P.L., Dalrymple B.P. (2009). Inferring the transcriptional landscape of bovine skeletal muscle by integrating co-expression networks. PLoS ONE.

[41] Fernández-Quintero M.L., El Ghaleb Y., Tuluc P., Campiglio M., Liedl K.R., Flucher B.E. (2021). Structural determinants of voltage-gating properties in calcium channels. Elife.

[42] Flucher B.E., Tuluc P. (2017). How and why are calcium currents curtailed in the skeletal muscle voltage-gated calcium channels?. J. Physiol..

[43] Gönczi M., Dienes B., Dobrosi N., Fodor J., Balogh N., Oláh T., Csernoch L. (2021). Septins, a cytoskeletal protein family, with emerging role in striated muscle. J. Muscle. Res. Cell Motil..

[44] Wang X., Fei F., Qu J., Li C., Li Y., Zhang S. (2018). The role of septin 7 in physiology and pathological disease: A systematic review of current status. J. Cell. Mol. Med..

[45] Zhang Y., Cui D., Huang M., Zheng Y., Zheng B., Chen L., Chen Q. (2023). NONO regulates B-cell development and B-cell receptor signaling. FASEB J..

